# Insights on repetitive DNA behavior in two species of *Ctenus* Walckenaer, 1805 and *Guasuctenus* Polotow and Brescovit, 2019 (Araneae, Ctenidae): Evolutionary profile of H3 histone, 18S rRNA genes and heterochromatin distribution

**DOI:** 10.1371/journal.pone.0231324

**Published:** 2020-04-08

**Authors:** Matheus Pires Rincão, Antonio Domingos Brescovit, Ana Lúcia Dias

**Affiliations:** 1 Laboratório de Citogenética Animal, Departamento de Biologia Geral, CCB, Universidade Estadual de Londrina, Londrina, Brazil; 2 Laboratório Especial de Coleções Zoológicas, Instituto Butantan, São Paulo, Brazil; Tulane University Health Sciences Center, UNITED STATES

## Abstract

Ctenidae represents one of the most representative spider families in the tropical forests of Brazil. Its largest genus, *Ctenus*, has approximately 220 species out of the more than 520 Ctenidae species described, and several authors consider it polyphyletic. Chromosomal data are only available for four species of *Ctenus*, representing a large gap in the cytogenetic knowledge about the group. This study provided cytogenetic data on two *Ctenus* species and one *Guasuctenus* (previously described as *Ctenus*). All showed 2n♂ = 28 (26+X_1_X_2_0). *Guasuctenus longipes* presented two chromosome pairs containing 18S rDNA genes and *C*. *medius*, however *C*. *ornatus* showed only one chromosome pair with the 18S rDNA gene. Hybridization data using histone H3 probe indicated specific profiles: histone H3 genes were found in one chromosome pair in G. *longipes*, in three pairs in *C*. *medius*, and in four pairs in *C*. *ornatus*. Furthermore, supernumerary chromosomes were identified in *C*. *ornatus* presenting a meiotic behavior similar to that of sex chromosomes; and a trivalent was found in *C*. *medius*, formed by the association of one sex chromosome and an autosomal bivalent, indicating the importance of these events for the diversification of sex chromosomes in spiders. The C-banding pattern was similar between *C*. *medius* and *C*. *ornatus* with regard to the number and locations of heterochromatic bands, suggesting that heterochromatin amplification and dispersion, affect karyotypic evolution in the genus. Cytogenetic data showed similarity between *C*. *medius* and *C*. *ornatus*, and differentiation of *G*. *longipes* congruent with morphological data. Moreover, although more comparative analyses are needed to specify composition of the dispersed heterochromatin in *Ctenus*, the mapping of heterochromatic bands provided insights about the evolution of the karyotypes in this genus.

## Introduction

Ctenidae are one of the best-known spider families worldwide and contain species of medical interest, such as those of the genus *Phoneutria*, popularly known as “banana spider” or “armed spider”. This family has 48 genera and approximately 520 species [1] and is usually found in tropical forests. Based on morphological and molecular data, Ctenidae belong to the superfamily Lycosoidea, together with Oxyopidae, Thomisidae, Psechridae, Trechaleidae, Pisauridae, and Lycosidae [2, 3]; it constitutes the third largest clade of this superfamily.

The genus *Ctenus*, proposed by Walckenaer in 1805, contains more than 200 currently known species, corresponding to approximately 40% of the number of species in Ctenidae [1], and it is considered polyphyletic [3, 4, 5, 6, 7]. Polotow and Brescovit [7], in a study involving 32 species of *Ctenus*, showed that for genus to be considered monophyletic, 19 species should be transferred to other genera. Regarding these results, *C*. *longipes* was recently transferred to *Guasuctenus* Polotow and Brescovit [8], that presents today two Neotropical species [1].

Only four species of *Ctenus* have been karyotyped until date, representing less than 2% of the species described in the genus, indicating a significant gap in the cytogenetic knowledge about the group. *Ctenus indicus* (Gravely 1931), *C*. *medius* Kesselring 1891, *C*. *ornatus* (Kesselring 1877), and *Ctenus* sp. have 28 acrocentric/telocentric chromosomes (2n♂ = 26 + X_1_X_2_0) [9, 10, 11]. Of these four species, three have data on nucleolar organizer regions (NORs) evidenced by either impregnation with silver nitrate (*C*. *ornatus* [9] and *C*. *indicus* [10]) or fluorescence *in situ* hybridization (FISH) (*C*. *ornatus* and *C*. *medius* [11]). *Ctenus indicus* is the only species with multiple NORs.

Analysis of heterochromatin distribution was also important for characterization of *Ctenus* species, since only *C*. *ornatus* presented interstitial heterochromatic blocks [11], while the other species of the genus presented centromeric/pericentromeric regions throughout the chromosome complement, with terminal blocks on the long arm of some chromosomes. The latter profile was observed by Rincão et al. [11] in other species of the family, including *Enoploctenus cyclothorax* (Bertkau 1880), *Viracucha andicola* (Simon, 1906) and *Phoneutria nigriventer* (Keyserling, 1891).

The scarcity of chromosomal data in other species of the genus limits establishing evolutionary relationships between karyotypes. The objective of this study was to provide for the first time the cytogenetic description of *G*. *longipes* and new populations of *C*. *medius* and *C*. *ornatus* to investigate, using chromosomal markers, the events that may have occurred during karyotype differentiation in these species and to correlate the results with cytogenetic and phylogenetic data available for this group.

## Material and methods

### Sample collection

Males and females, adults and juveniles, of three ctenid species, *G*. *longipes*, *C*. *medius* and *C*. *ornatus*, were collected in two conservation units, Parque Estadual Mata dos Godoy (PEMG) (W 51° 14' 31,86", S 23° 26' 29,37") and Parque Nacional do Superagui (PNS) (W 48° 13' 51,9", S 25° 27' 14,40"), state of Paraná, Brazil, as showed in **Table 1**. Sampling was performed with the permission of Instituto Brasileiro do Meio Ambiente e dos Recursos Naturais Renováveis (IBAMA/SISBIO) license number 42728–5. The specimens were stored in ethanol 70% and deposited in the arachnological collection of the Laboratório Especial de Coleções Zoológicas, Intituto Butantan (IBSP, curator AD Brescovit), São Paulo/SP, Brazil (**S1 Table**).

**Table 1:** Species analyzed and collection sites.

Species	Sample		Collection Sites
	♂	♀	
*Guasuctenus longipes*	12	2	PNS
	3	-	PEMG
*Ctenus medius*	12	2	PNS
	10	-	PEMG
*Ctenus ornatus*	7	2	PNS
	17	1	PEMG

PNS = Parque Nacional do Superagui Guaraqueçaba-PR; PEMG = Parque Estadual Mata dos Godoy, Londrina-PR.

### Cytogenetic analyses

Chromosomal preparations followed the protocol described by Araujo et al. [12], using testicles, ovaries and intestinal ducts. To assembly of the slides the tissue was treated and macerated in 60% acetic acid, then the slide was heated in a metal plate at 40° C. The diploid number was determined by counting 30 mitotic or meiotic cells. Chromosomes was classified according to Levan et al. [13]. C-banding followed Sumner [14], with the modifications proposed by Lui et al [15]: slides were stained with propidium iodide and photographed in gray scale. Staining with base-specific fluorochromes Chromomycin A3 (CMA3) and 4’, 6-diamidino-2-phenilindole (DAPI) followed the protocol described by Schweizer [16].

Chromosomal measurements for assembling karyotypes were performed in around 10 mitotic metaphases, using the MicroMeasure software, version 3.3 [17]. Sex chromosome systems were defined by comparing the mitotic metaphase measures and the X_1_:X_2_ size ratio in meiosis. For that were measured 30 meiotic cells. To determine the frequency of supernumerary chromosomes were analyzed 30 meiotic and mitotic cells.

### Fluorescence *In Situ* Hybridization (FISH)

FISH was performed according to Schwarzacher and Heslop-Harrison [18], 18S rDNA probes were obtained from *Ctenus ornatus* by Rincão et al. [11], amplified from the plasmid DNA by PCR with the M13 primer pair, labeled with the Biotin-Nick Translation Mix (Roche) kit and detected with Avidin-FITC (Invitrogen). Histone H3 probes were obtained by amplifying the genomic DNA of *C*. *ornatus*, with the aid of primers described by Colgan et al. [19]. The obtained fragments were cloned using the TOPO TA Cloning Kit (Invitrogen), in competent *Escherichia coli* strain (TOP 10). The insert was amplified by M13 primer pair for sequencing in ABIprism 3500 Genetic Analyzer (Aplied Biosystems), sequences were analyzed using BioEdit version 7.2.5 software [20]. A 370 bp consensus sequence was obtained, which was aligned on GenBank to confirm homology to other previously described histone H3 sequences for spiders. H3 probes were then labeled by PCR, using the M13 primer pair, with the Cy3 dUTP (GE Healthcare).

FISH slides were counterstained with DAPI and analyzed on the Leica DM2000 epifluorescence microscope equipped with a Moticam Pro 282B digital camera. The images were captured using Motic Images Advanced software, version 3.2.

## Results

### Guasuctenus longipes

**Fig 1:**
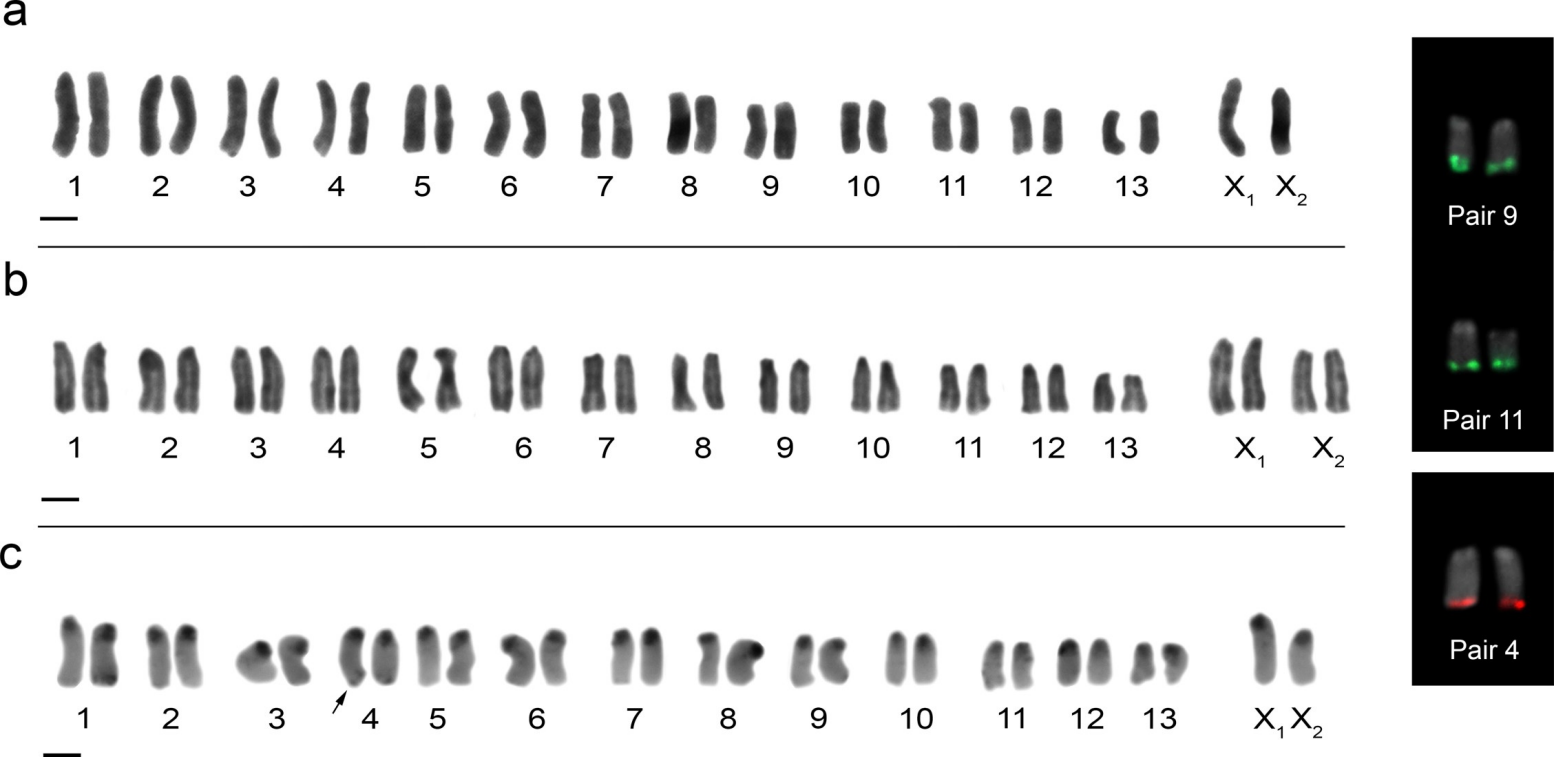
Male and female karyotypes of *G*. *longipes* with conventional staining Giemsa (**a**, **b**, respectively); male karyotype after C banding (**c**), arrow points to the terminal heterochromatic blocks on the long arm of pair 4. Inset box shows chromosome pairs with 18S rDNA (green) and histone H3 (red). Scale bar = 10 μm.

**Fig 2:**
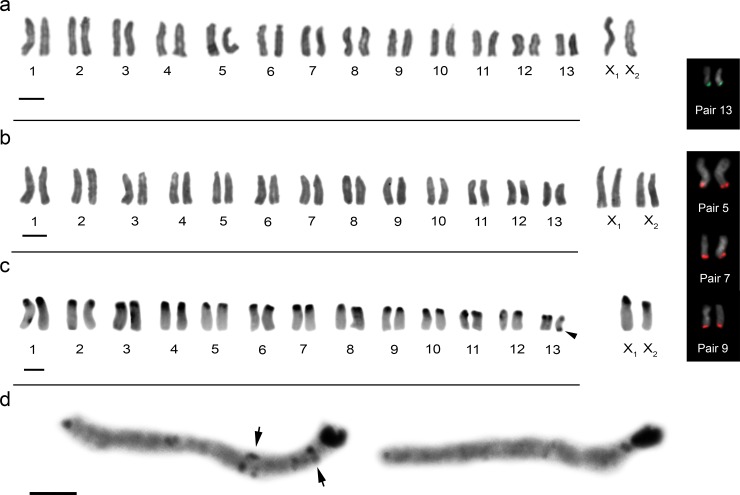
Male and female karyotypes of *C*. *medius* with conventional staining Giemsa (**a, b,** respectively); male karyotype after C banding (**c**) arrowhead points the terminal heterochromatic block on the long arm of one chromosome of the pair 13; two bivalents in pachytene after C-banding, arrows show interstitial heterochromatin in one of them (**d**). Inset box shows chromosome pairs with 18S rDNA (green) and histone H3 (red). Scale bar = 10 μm.

*Guasuctenus longipes* showed 2n = 28 (26 + X_1_X_2_0) in males (Fig 1A) and 2n = 30 (26 + X_1_X_1_X_2_X_2_) in females (Fig 1B). All chromosomes were acrocentric and the autosomal complement gradually decreasing in size. The X_1_:X_2_ size ratio obtained of meiotic cells showed that X_2_ sex chromosome corresponds to 79% of the X_1_ length. Therefore, the X_1_ and X_2_ sex chromosomes corresponds, respectively, to the largest and medium-sized chromosomes of the complement (Fig 1A and 1B). FISH technique indicated chromosomal pairs 9 and 11 carrying the 18S rDNA genes and pair 4 carrying the histone H3 genes (Fig 1, boxes), all these sites were present in the terminal region of the long arm of the chromosomes.

C-banding technique indicated the presence of heterochromatic blocks in the centromeric region of all chromosomes and smaller blocks in the terminal region of the long arm of pair 4 (Fig 1C). Fluorochrome staining showed regions DAPI^-^ and CMA3^+^ (Fig 5B), which were coincident with heterochromatic blocks.

Meiotic analyses in G. *longipes* indicated isopycnotic sex chromosomes in pachytene cells in females (S1A Fig). In males, sex chromosomes showed positive heteropycnosis and parallel association in the early stages of meiosis (S1B–S1D Fig). Metaphase II and anaphase II cells in males showed 13 or 15 chromosomes, confirming the X_1_X_2_0 sex chromosome system (SCS) (S1E and S1F Fig). In addition, satellites were found in association at the stage of diplotene in homologous and non-homologous chromosomes, of all specimens collected in the Parque Nacional de Superagui (PNS) (S1G and S1H Fig) and coincided with 18S rDNA sequences (pairs 9 and 11) (S1G and S1H Fig -box).

### Ctenus medius

*Ctenus medius* exhibited 2n = 28 (26 + X_1_X_2_0) in males (Fig 2A) and 2n = 30 (26 + X_1_X_1_X_2_X_2_) in females (Fig 2B). All chromosomes were acrocentric and the autosomal complement gradually decreasing in size. The X_1_:X_2_ size ration obtained of meiotic cells showed that X_2_ sex chromosome corresponds to 90% of the X_1_ chromosome length. Therefore, the X_1_ and X_2_ sex chromosomes were identified as the largest chromosomes of the diploid set (Fig 2A and 2B). FISH technique indicated chromosome pair 13 carrying 18S rDNA genes and pairs 5, 7 and 9 carrying histone H3 genes (Fig 2, boxes); all sites were present in the terminal region of the long arm of the chromosomes.

C-banding analyses showed that large heterochromatic blocks were in the centromeric region of all chromosomes (Fig 2C) and in the terminal region of one chromosome of pair 13. However, cells in the pachytene indicated some bivalent had small heterochromatin dots in interstitial regions (Fig 2D). CMA_3_ and DAPI staining showed that heterochromatic regions are GC-rich (Fig 5C and 5D).

**Fig 3:**
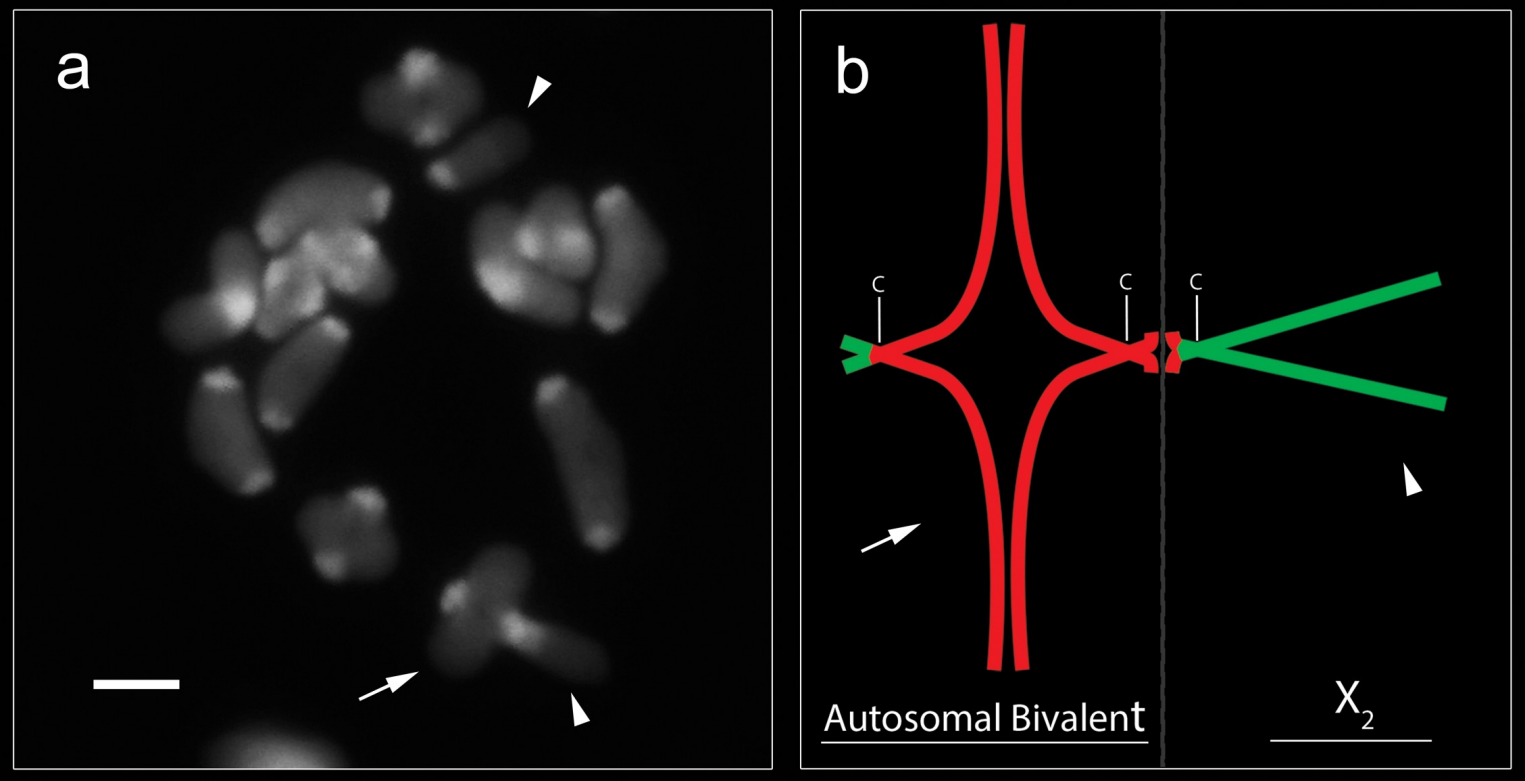
Diakinesis cell of *Ctenus medius* after C-banding. **c =** centromere region. (**a**), arrowheads show sex chromosomes and arrow show an autosomal bivalent which, in association with the X_2_ sex chromosome forms the trivalent; graphical representation of trivalent formation in diakinesis, showing pairing in the region of short arm between the autosomal bivalent and the X_2_ sex chromosome, suggesting a possible translocation (**b**). Scale bar = 10 μm.

Meiotic analyses in *C*. *medius* indicated isopycnotic sex chromosomes in pachytene cells of females (S2A Fig). In males, sex chromosomes exhibited positive heteropycnosis and parallel association in the early stages of meiosis (S2B–S2D Fig). Metaphases II showed cells with 13 or 15 chromosomes, confirming X_1_X_2_0 SCS (S2E and S2F Fig). In one specimen from the population of Parque Estadual Mata dos Godoy (PEMG), we observed a trivalent formed from the association between the sex chromosome X_2_ and an autosomal bivalent (S2G and S2H Fig). C-banding analyses indicated the pairing of an autosomal bivalent with sex chromosome X_2_ in the short arm (Fig 3A). A graphical representation of the trivalent is shown in Fig 3B.

### Ctenus ornatus

**Fig 4:**
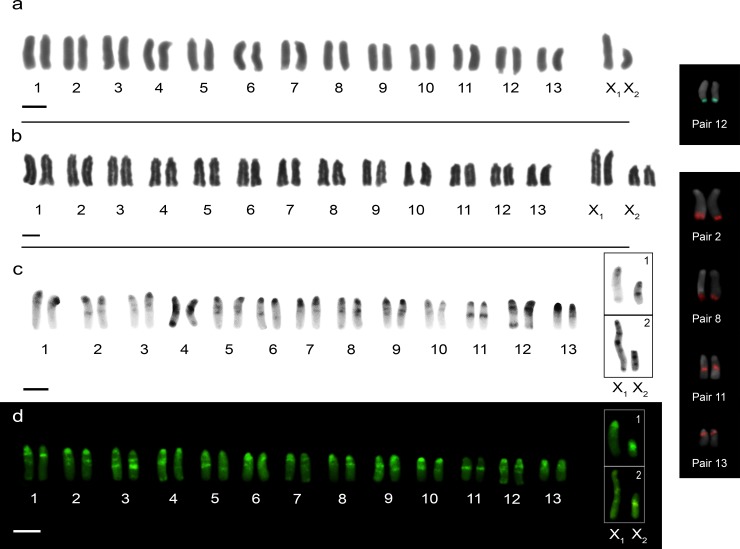
Male and female karyotypes of *Ctenus ornatus* with conventional staining Giemsa (**a, b,** respectively); male karyotype after C-banding (**C**) and staining with fluorochrome CMA_3_ (**d**), inset boxes show the sex chromosome pair of the Parque Estadual Mata dos Godoy (**c1**, **d1**) and Parque Nacional do Superagui (**c2**, **d2**): note the difference in X_1_ sex chromosome banding pattern between populations. Inset box shows chromosome pairs with 18S rDNA (green) and Histone H3 (red). Scale bar = 10 μm.

*Ctenus ornatus* showed 2n = 28 (26 + X_1_X_2_0) in males (Fig 4A) and 2n = 30 (26 + X_1_X_1_X_2_X_2_) in females (Fig 4B). All chromosomes were acrocentric and the autosomal complement gradually decreasing in size. The X_1_:X_2_ size ratio obtained of meiotic cells showed that X_2_ sex chromosome corresponds to 75% of the X_1_ chromosome length. However, additional analyses with C-banding, evidenced the X_1_ and X_2_ sex chromosomes as the largest and the smallest chromosomes in the complement (Fig 4A, 4B and 4C). In mitotic metaphase cells, secondary constrictions were observed in the terminal region of the long arm of pair 12 (Fig 4A). FISH technique demonstrated the presence of 18S rDNA genes in the terminal region of the long arm of pair 12 (Fig 4, box), coincident with the secondary constriction (Fig 4A) and the presence of histone H3 genes in pairs 2, 8, 11 and 13, the last two with interstitial markings (Fig 4).

**Fig 5:**
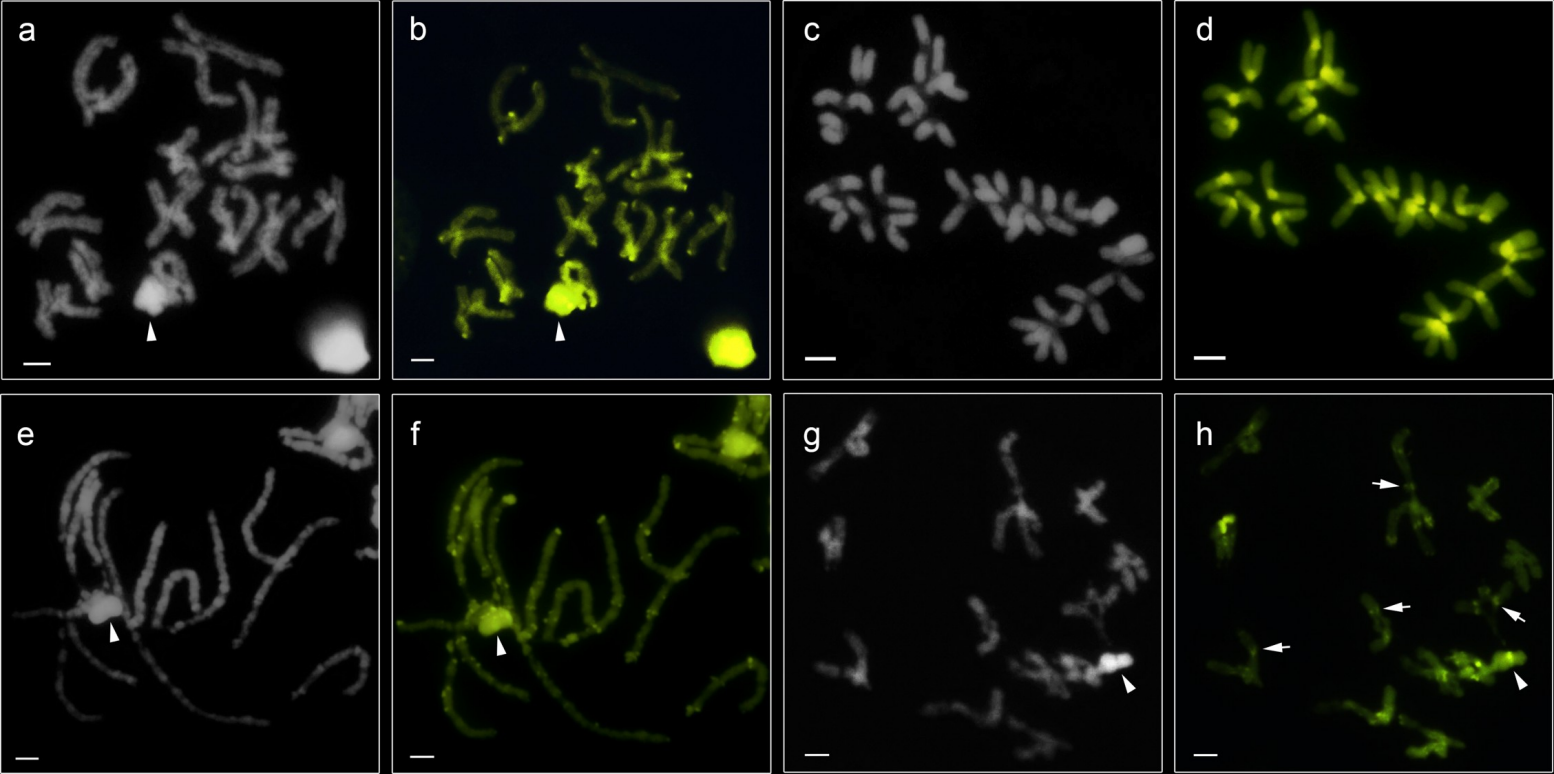
Meiotic cells after staining with base-specific fluorochromes DAPI (**a, c, e, g**) and CMA3 (**b, d, f, h**). Arrowheads point sex chromosomes. Diplotene cells of *Guasuctenus longipes* (**a, b**); Metaphase II cells of *C*. *medius* (**c**, **d**); Pachytene cells (**e**, **f**) and diplotene cells (**g**, **h**) of *C*. *ornatus*: arrows (**h**) point to interstitials blocks. Scale bar = 10 μm.

C-banding technique evidenced heterochromatic blocks in the centromeric, interstitial and terminal regions of the chromosomes. However, the profile of the X_1_ sex chromosome differed between the two populations (Fig 4C and 4D). CMA_3_ and DAPI staining showed the presence of GC-rich regions, CMA_3_^+^ and DAPI^-^, coincident with the C-banding pattern (Figs 4D and 5E–5H).

Meiotic analyses in *C*. *ornatus* evidenced isopycnotic sex chromosomes in pachytene cells of females (S3A Fig). In males, sex chromosomes showed positive heteropycnosis in the early stages of meiosis (S3B and S3C Fig). Metaphases II showed cells with 13 and 15 chromosomes, confirming the X_1_X_2_0 SCS (S3D Fig). In the PNS population, we observed two specimens with one supernumerary chromosome (S3E–S3G Fig), with approximately 50% of the cells in each one, and one specimen with two supernumerary chromosomes (S3H Fig), in 53% of the cells analyzed. These chromosomes showed positive heteropycnosis (S3E Fig) and behavior similar to the sex chromosomes (S3F and S3H Fig)

**Fig 6:**
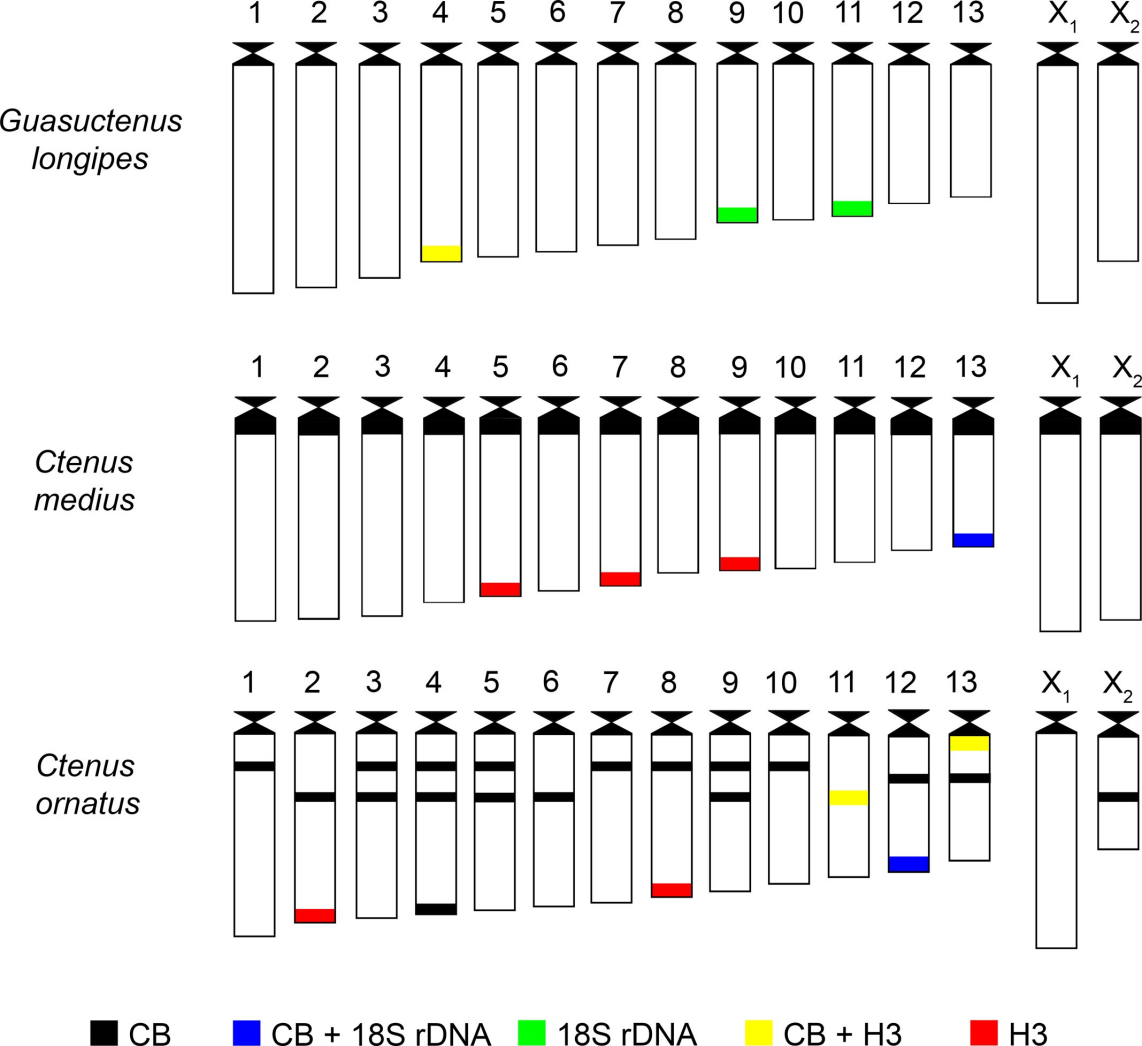
Representative idiogram of *Guasuctenus*. *longipes*, *Ctenus medius* e *C*. *ornatus* showing different banding: C-Banding (CB), 18S rDNA and histone H3 sites and regions where the heterochromatin blocks are coincident with the 18 rDNA or histone H3 sites.

An idiogram of the distribution of heterochromatin, 18S rRNA, and histone H3 genes in the karyotype of *G*. *longipes*, *C*. *medius*, and *C ornatus* is shown in Fig 6.

## Discussion

### Karyotypic analysis

The karyotypes of *G*. *longipes*, first described, *C*. *medius* and *C*. *ornatus* showed a conserved pattern, similar to the ancestral karyotype described for the superfamily Lycosoidea [21, 22]. This karyotype with 2n♂ = 26 + X_1_X_2_0, is present in all *Ctenus* species and varies only in the size ratio of the sex chromosomes [9, 10, 11, 23]. The divergence about X_1_:X_2_ size ratio and the definition of the X_2_ sex chromosome in *C*. *ornatus*, may be explained by higher condensation of the sex chromosomes in meiosis, as discussed by Dolejš et al. [21]. Therefore, the karyotypic structure is well conserved in *Ctenus*, indicating the need to evaluate other characteristics to differentiate the species of the genus from a cytogenetic point of view.

A common feature in spider karyotype is the presence of secondary constrictions [21, 22, 24], which had been described in Ctenidae only in *Nothoctenus* sp. and *Ctenus* sp. [9]. In *C*. *ornatus* and G. *longipes*, secondary constrictions were shown to be associated to 18S rDNA, and this type of association is recurrent in several animal groups, such as the grasshopper *Abracris flavolineata* De Geer 1773 [25] and the fish *Loricariichthys platymetopon* Isbrücker and Nijssen, 1979 [26].

Araujo et al. [9] also reported the occurrence of one supernumerary chromosome in a male specimen of *C*. *ornatus* with meiotic behavior of univalent similar to that in sex chromosomes. Since this system is recurrent in spiders, the authors proposed that this may be an origin event of X_1_X_2_X_3_0 SCS [9, 27, 28, 29]. This same event may be occurring in the cells of the analyzed specimens in this study. And the presence of two supernumerary chromosomes in other specimen may demonstrate the conversion for a X_1_X_2_X_3_X_4_0 SCS in Ctenidae for the first time, thus occurring systems involving neo-X_3_ and neo-X_4_ sex chromosomes.

The occurrence of supernumerary can be considered recurrent in *C*. *ornatus* because the distance from the region sampled in this study to the region sampled by Araujo et al. [9] was >300 km. However, further analyses are necessary to determine the frequency of these chromosomes in different populations of this species and elucidate the mechanisms involved in its formation.

A very striking feature of the karyotype of all analyzed Lycosoidea species is the difference in the size of the sex chromosomes, which was also observed among the three species analyzed here, in the ratio of size X_1_: X_2_. This chromosome variation between species may be the result of translocations between autosomes and sex chromosomes, as discussed earlier by Dolejš et al. [21], for Lycosidae species. Probably, the amount of heterochromatin is not necessarily involved in this polymorphism because in *C*. *ornatus*, despite the very evident heterochromatic bands, the sex chromosome X_2_ was identified as the smallest complement chromosome. Therefore, the presence of a trivalent in a specimen of *C*. *medius* from PEMG, involving the association of an autosomal bivalent with a sex chromosome, reinforces the hypothesis of Dolejš et al. [21] that translocations are one of the main mechanisms responsible for the size differentiation of sex chromosomes in this superfamily.

### Inferences about the distribution of repetitive DNA in Cteninae

Repetitive DNA sequences in the terminal regions of the chromosomes are common in Ctenidae, including heterochromatic regions and 18S rRNA genes [10, 11]. Dolejs et al. [21] suggest that NORs in the terminal region of the long arm of autosomal chromosomes constitute a plesiomorphic condition in Araneomorphae, which appears to apply to Ctenidae, even in subfamilies such as Acantheinae and Acanthocteninae [11], which have ancestral morphological traits [7].

*Guasuctenus longipes* (present study), *C*. *indicus* [10] and *Phoneutria nigriventer* [11] were the only species in Cteninae that demonstrated to have more than one chromosomal pair carrying 18S rDNA genes. In this respect, *C*. *medius* and *C*. *ornatus* had a similar profile, in addition to having an increase in the number of histone H3 genes and heterochromatin distribution; the latter was significant in *C*. *medius* owing to the size of the heterochromatic blocks and in *C*. *ornatus* because of the number and the distribution profile of these blocks (Fig 6).

Results of a comparative analysis of the most recent cytogenetic and phylogenetic data for Cteninae indicated that the close relationship between *C*. *medius* and *C*. *ornatus* found in the present study, and the very different karyotype profile for *G*. *longipes* (previously described as *C*. *longipes*), are well supported by the morphological analysis of Polotow and Brescovit [7], who studied 32 species of *Ctenus* and concluded that the genus was polyphyletic. These authors indicated that *C*. *medius* was grouped in a clade (Clade F) together with *C*. *ornatus*, *C*. *vehemens*, *C*. *fernandae* and *C*. *paubrasil*, among other species, whereas *G*. *longipes* belonged to a separate clade (Clade K) and was more phylogenetically related to species of the genera *Parabatinga* and *Isoctenus*.

The presence of interstitial heterochromatin bands in *C*. *ornatus* may appear to be an apomorphic feature of the species, but it is not unique to it. C-banding analyses in pachytene cells of *C*. *medius* indicated the presence of a few interstitial heterochromatic bands in some chromosomes, but not in mitotic metaphase, probably because of their high degree of condensation. Moreover, the colocalization of histone H3 and the heterochromatic bands, in specially the interstitial bands in *C*. *ornatus*, suggest a direct relationship of evolution between histone genes and heterochromatin in *Ctenus*.

Therefore, although cytogenetic data are not available for other species of the clade where *C*. *medius* and *C*. *ornatus* are grouped, it can be concluded that amplification and dispersion events of heterochromatin and repetitive DNA sequences, such as those originated by transposons and ectopic exchanges [30], were significant for the evolutionary history of the karyotypes of both species. The differences in the sex chromosome X_1_ between two *C*. *ornatus* populations suggest that these mechanisms continue to occur. Furthermore, the presence of interstitial histone H3 sites may be evidence that paracentric inversions also played a role in karyotype differentiation in this group of spiders.

This study, besides bringing the first physical mapping data of histone H3 in spiders and the first cytogenetic description in a new genus, shows the potential of chromosomal markers in the karyotype characterization in Araneae. In addition to histone genes, heterochromatic and 18S rDNA profiles showed a similarity between *C*. *medius* and *C*. *ornatus*, and a differentiation of *G*. *longipes* congruent with the morphological data.

## Supporting information

S1 Table Specimens separated by collection sites. PNS = Parque Nacional de Superagui; PEMG = Parque Estadual Mata dos Godoy. The numbers determine specimen voucher deposited in the arachnological collection of the Laboratório Especial de Coleções Zoológicas, Intituto Butantan (IBSP, curator AD Brescovit), São Paulo/SP, Brazil. (PDF 41 kb).
(PDF)

S1 Fig Female (**a**) and male (**b**-**h**) meiotic cells of *G*. *longipes* with conventional staining Giemsa. Arrowheads point sex chromosomes. Pachytene cells (**a**, **b**); diplotene (**c**); diakinesis (**d**); metaphase II (**e**); anaphase II (**f**); diplotene (**g**, **h**); arrows show associated satellites between homologous (**g**) and non-homologous chromosomes (**h**) in all individuals of the PNS population coinciding with 18S rDNA sites (boxes). (PDF 211 kb).
(PDF)

S2 Fig Female (**a**) and male (**b**-**h**) meiotic cells of *C*. *medius* with conventional staining Giemsa. Arrowheads point sex chromosomes. Pachytene cells (**a**, **b**); diakinesis cell (**c**); metaphase I cell (**d**); metaphases II cells, with 15 and 13 chromosomes (**e**, **f**, respectively); diakinesis (**g**) and pachytene cells (**h**), showing the trivalent observed in the individual of PEMG. (PDF 1312 kb).
(PDF)

S3 Fig Female (**a**) and male (**b**-**h**) meiotic cells of *C*. *ornatus* with conventional staining Giemsa. Arrowheads point sex chromosomes. Pachytene cells (**a**, **b**); diakinesis cell (**c**); metaphases II cells; pachytene, diakinesis and metaphases II cells (**e**, **f**, **g**, respectively, showing one supernumerary chromosome (**S**), totally heteropicnotic (**e**); diakinesis cell evidencing two supernumerary chromosomes (**S**_**1**_ and **S**_**2**_) with similar behavior to sex chromosomes. (PDF 215 kb).
(PDF)
