# Supplementary material for: Insights on repetitive DNA behavior in two species of Ctenus Walckenaer, 1805 and Guasuctenus Polotow and Brescovit, 2019 (Araneae, Ctenidae): Evolutionary profile of H3 histone, 18S rRNA genes and heterochromatin distribution
Source: PLoS One. 2020 Apr 8;15(4):e0231324. doi: 10.1371/journal.pone.0231324 (PMC7141658; doi:10.1371/journal.pone.0231324)
Supplement: S1 Table — PNS = Parque Nacional de Superagui; PEMG = Parque Estadual Mata dos Godoy. The numbers determine specimen voucher deposited in the arachnological collection of the Laboratório Especial de Coleções Zoológicas, Intituto Butantan (IBSP, curator AD Brescovit), São Paulo/SP, Brazil. (PDF 41 kb). (PDF) [file pone.0231324.s001.pdf]

Table S1. Specimens separated by collection sites. PNS = Parque Nacional do Superagui; PEMG = Parque Estadual Mata dos Godoy. The numbers determine specimen voucher deposited in the arachnological collection of the Laboratório Especial de Coleções Zoológicas, Intituto Butantan (IBSP, curator AD Brescovit), São Paulo/SP, Brazil.

Ctenus medius		*Ctenus ornatus*		*Guasuctenus longipes*	
PEMG	PNS	PEMG	PNS	PEMG	PNS
215931	217942	215932	215943	215885	215926
215936	217943	217937	215944	215916	215937
217920	215942	217938	215939	215916	215923
217927	217931	217939	217936		215945
217925	217930	217940	217935		215935
217922	217934	217941	217933		215934
217924	215891	217926	215910		215930
217954	215925	217944	216074		215866
217955	215892	217945	216103		216116
217957	217932	217946			216105
	217921	217948			216086
	217759	217950			214465
	216084	217958			216117
	216083	217879			217761
	217756	217879			
	216119	217915			
		217916			
		217917			
