# Supplementary material for: Insights on repetitive DNA behavior in two species of Ctenus Walckenaer, 1805 and Guasuctenus Polotow and Brescovit, 2019 (Araneae, Ctenidae): Evolutionary profile of H3 histone, 18S rRNA genes and heterochromatin distribution
Source: PLoS One. 2020 Apr 8;15(4):e0231324. doi: 10.1371/journal.pone.0231324 (PMC7141658; doi:10.1371/journal.pone.0231324)
Supplement: S1 Fig — Female (a) and male (b-h) meiotic cells of G. longipes with conventional staining Giemsa. Arrowheads point sex chromosomes. Pachytene cells (a, b); diplotene (c); diakinesis (d); metaphase II (e); anaphase II (f); diplotene (g, h); arrows show associated satellites between homologous (g) and non-homologous chromosomes (h) in all individuals of the PNS population coinciding with 18S rDNA sites (boxes). (PDF 211 kb). (PDF) [file pone.0231324.s002.pdf]

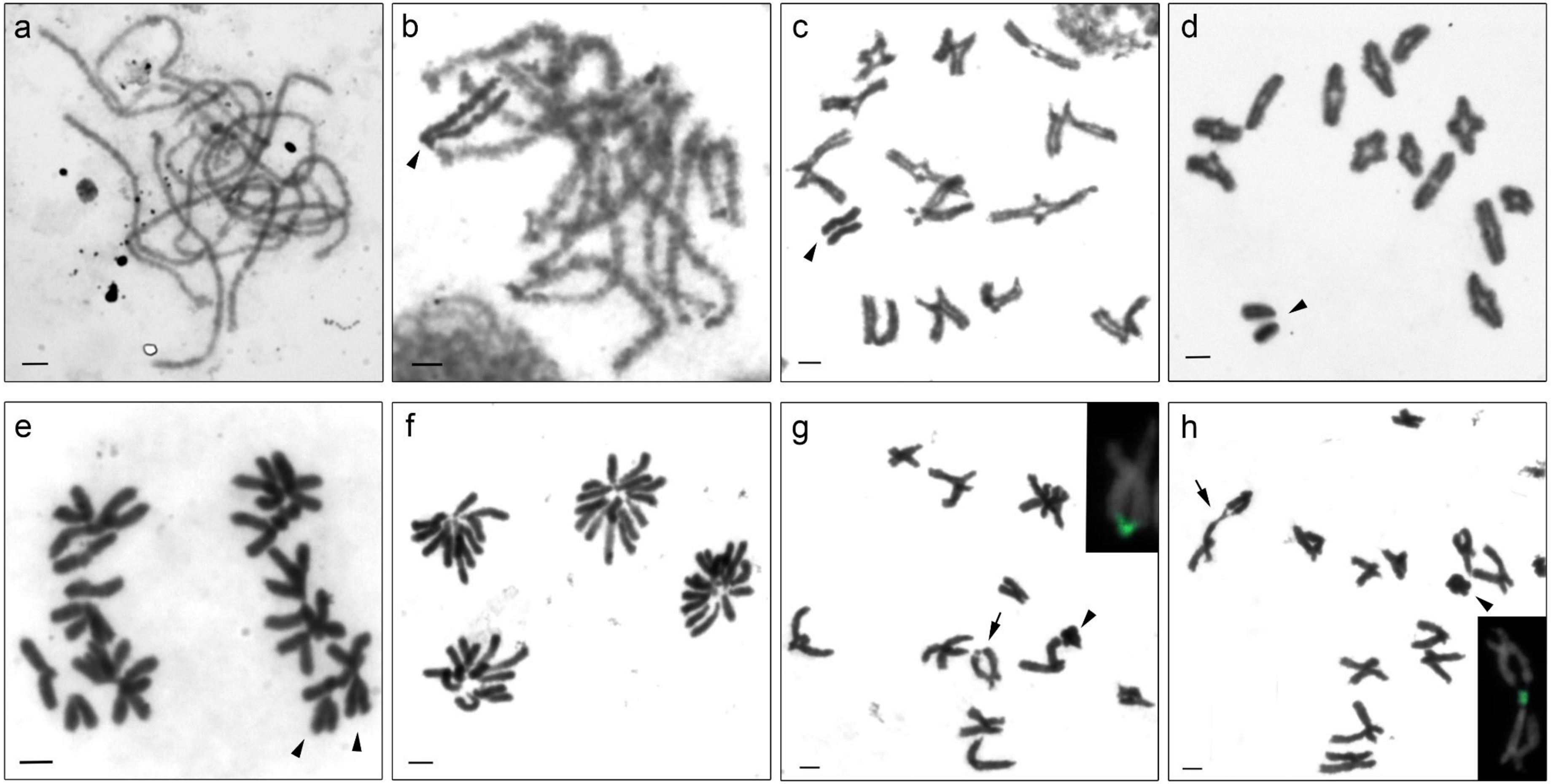

Fig. S1 Female (**a**) and male (**b-h**) meiotic cells of *C. longipes* with conventional staining Giemsa. Arrowheads point sex chromosomes. Pachytene cells (**a**, **b**); diplotene (**c**); diakinesis (**d**); metaphase II (**e**); anaphase II (**f**); diplotene (**g**, **h**); arrows show associated satellites between homologous (**g**) and non-homologous chromosomes (**h**) in all individuals of the Parque Nacional do Superagui population coinciding with 18S rDNA sites (boxes). Scale bar = 10 µm.
