# Supplementary material for: Insights on repetitive DNA behavior in two species of Ctenus Walckenaer, 1805 and Guasuctenus Polotow and Brescovit, 2019 (Araneae, Ctenidae): Evolutionary profile of H3 histone, 18S rRNA genes and heterochromatin distribution
Source: PLoS One. 2020 Apr 8;15(4):e0231324. doi: 10.1371/journal.pone.0231324 (PMC7141658; doi:10.1371/journal.pone.0231324)
Supplement: S2 Fig — Female (a) and male (b-h) meiotic cells of C. medius with conventional staining Giemsa. Arrowheads point sex chromosomes. Pachytene cells (a, b); diakinesis cell (c); metaphase I cell (d); metaphases II cells, with 15 and 13 chromosomes (e, f, respectively); diakinesis (g) and pachytene cells (h), showing the trivalent observed in the individual of PEMG. (PDF 1312 kb). (PDF) [file pone.0231324.s003.pdf]

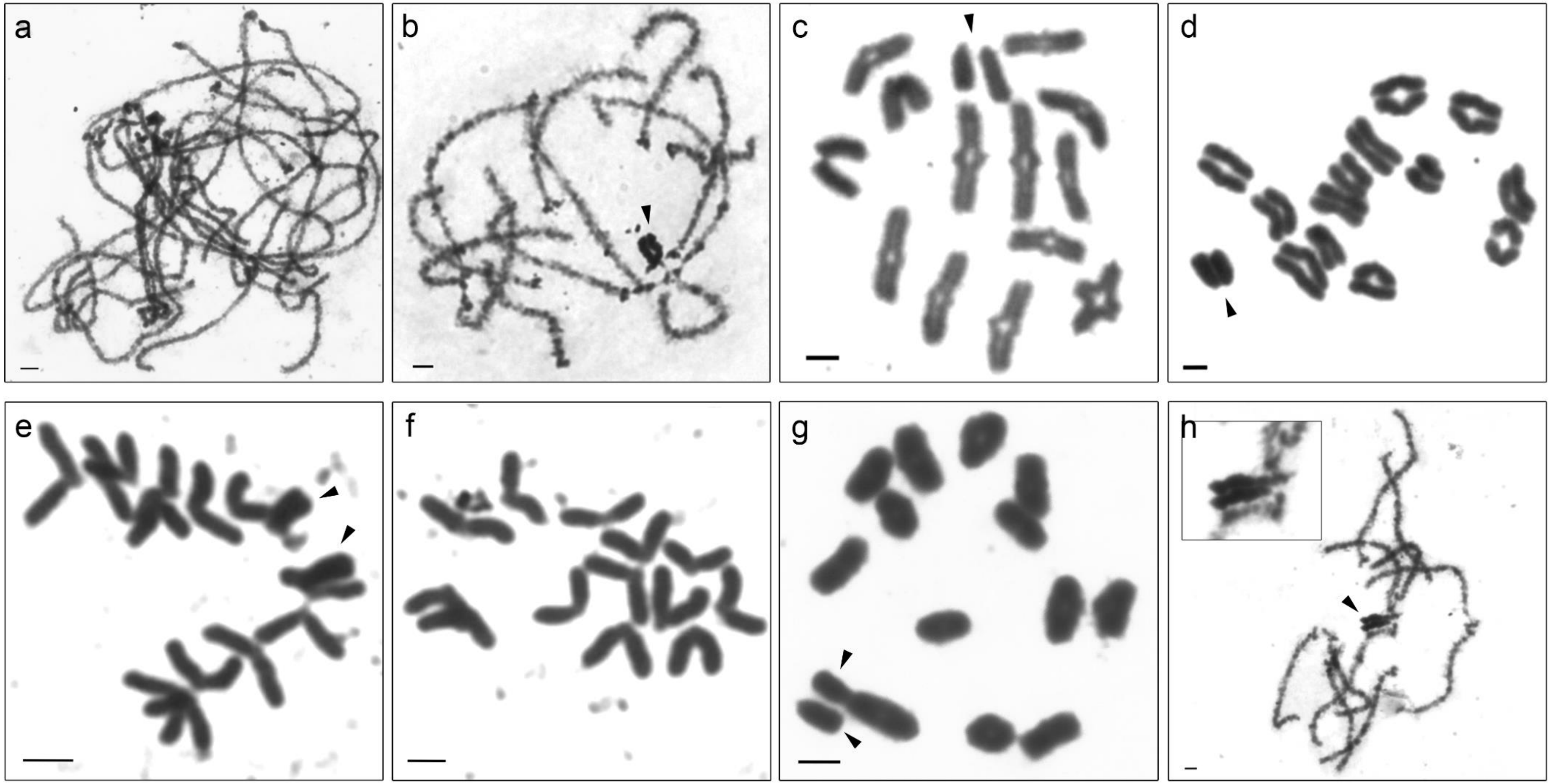

Fig. S2 Female (**a**) and male (**b**-**h**) meiotic cells of *C. medius* with conventional staining Giemsa. Arrowheads point sex chromosomes. Pachytene cells (**a**, **b**); diakinesis cell (**c**); metaphase I cell (**d**); metaphases II cells, with 15 and 13 chromosomes (**e**, **f**, respectively); diakinesis (**g**) and pachytene cells (**h**), showing the trivalent observed in the individual of Parque Estadual Mata dos Godoy. Scale bar = 10 μm.
