# Supplementary material for: Insights on repetitive DNA behavior in two species of Ctenus Walckenaer, 1805 and Guasuctenus Polotow and Brescovit, 2019 (Araneae, Ctenidae): Evolutionary profile of H3 histone, 18S rRNA genes and heterochromatin distribution
Source: PLoS One. 2020 Apr 8;15(4):e0231324. doi: 10.1371/journal.pone.0231324 (PMC7141658; doi:10.1371/journal.pone.0231324)
Supplement: S3 Fig — Female (a) and male (b-h) meiotic cells of C. ornatus with conventional staining Giemsa. Arrowheads point sex chromosomes. Pachytene cells (a, b); diakinesis cell (c); metaphases II cells; pachytene, diakinesis and metaphases II cells (e, f, g, respectively, showing one supernumerary chromosome (S), totally heteropicnotic (e); diakinesis cell evidencing two supernumerary chromosomes (S1 and S2) with similar behavior to sex chromosomes. (PDF 215 kb). (PDF) [file pone.0231324.s004.pdf]

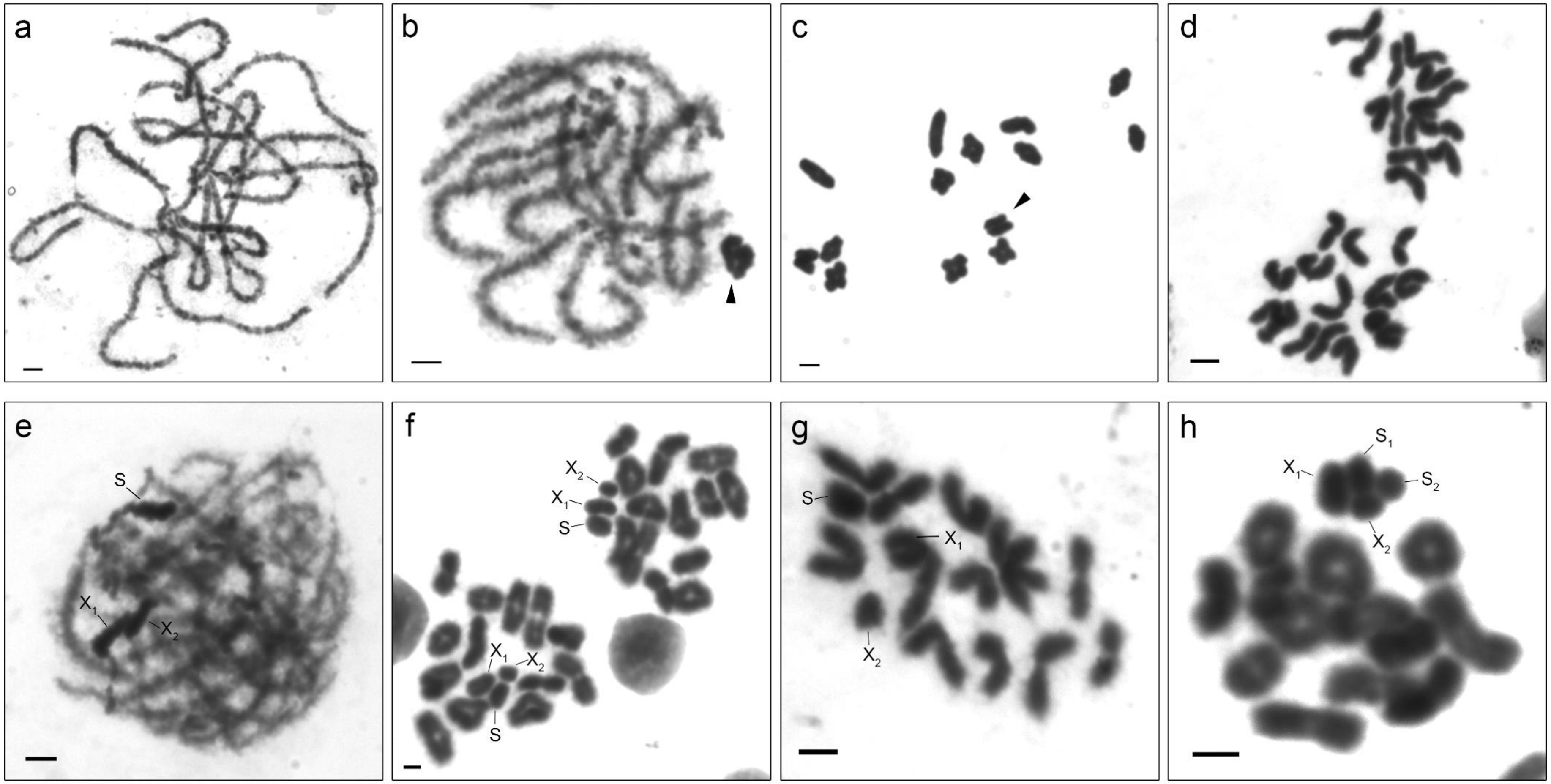

Fig. S3 Female (**a**) and male (**b-h**) meiotic cells of *C. ornatus* with conventional staining Giemsa. Arrowheads point sex chromosomes. Pachytene cells (**a**, **b**); diakinesis cell (**c**); metaphases II cells; pachytene, diakinesis and metaphases II cells (**e**, **f**, **g**, respectively, showing one supernumerary chromosome (**S**), totally heteropicnotic (**e**); diakinesis cell evidencing two supernumerary chromosomes (**S_1** and **S_2**) with similar behavior to sex chromosomes. Scale bar = 10 µm.
